# A qualitative evaluation of the challenges in management for patients with chronic diseases during disasters in Iran

**DOI:** 10.5249/jivr.v15i2.1767

**Published:** 2023-07

**Authors:** Elham Ghazanchaei, Kiyoumars Allahbakhshi, Davoud Khorasani-Zavareh, Javad Aghazadeh-Attari, Iraj Mohebbi

**Affiliations:** ^*a*^ Social Determinants of Health Research Center Urmia University of Medical Sciences, Urmia, Iran.; ^*b*^ Department of Health in Emergencies and Disasters, School of Public Health, Tehran University of Medical Sciences, Tehran, Iran.; ^*c*^ Skull Base Research Center, Loghman Hakim Hospital, Shahid Beheshti University of Medical Sciences, Tehran, Iran.; ^*d*^ Department of Health in Emergencies and Disasters, School of Public Health and Safety, Shahid Beheshti University of Medical Sciences, Tehran, Iran.

**Keywords:** Health literacy, Health promotion, Integrated management, Physical and psychosocial health, Risk perception, Service barriers

## Abstract

**Background:**

Iran’s health care system faces significant challenges in managing the growing burden of non-communicable diseases, and these are exacerbated during the frequent natural disasters. The current study was designed to understand challenges in providing healthcare services to patients with diabetes and chronic respiratory diseases during such crisis periods.

**Methods:**

The conventional content analysis was used in this qualitative study. Participants included 46 patients with diabetes and chronic respiratory diseases, and 36 stakeholders with knowledge and experience in disasters. Data collection was carried out employing semi-structured interviews. Data analysis was performed using Graneheim and Lundman method.

**Results:**

Four major challenges in providing care to patients with diabetes and chronic respiratory diseases during natural disasters include integrated management, physical, psychosocial health, health literacy and the behavior and barriers to healthcare delivery.

**Conclusions:**

Developing countermeasures against medical monitoring system shutdown in order to detect medical needs and problems faced by chronic disease patients including those with diabetes and chronic obstructive pulmonary disease (COPD), is essential in preparedness for future disasters. Developing effective solutions may result in improved preparedness and better planning of diabetic and COPD patients for disasters.

## Introduction

Non-communicable diseases (NCDs) are among major health challenges and the leading cause of deaths in the twenty first century.^1,2^ The burden of NCDs is increasing worldwide particularly in developing countries.^3^ Seven out of ten deaths in developing countries are caused by NCDs^4-7^ NCDs such as cancers, diabetes, cardiovascular and chronic respiratory diseases are among main global threats that have caused major public health challenges around the world. Therefore, the World Health Organization (WHO) has estimated the number of deaths from chronic diseases to increase from 38 million (68 percent) in 2012 to 55 million in 2030.^8-10^ Half of the deaths are in Asian countries constituting 47 percent of the global burden of disease.^4,8,10,11-13^

Among which, chronic obstructive pulmonary disease (COPD) is one major public health issue causing deaths worldwide.^14^ More than 90 percent of deaths from COPD occur in lower to middle income countries.^15^ COPD is predicted by WHO to become the third leading cause of death by 2030. The prevalence rate of the disease varies in different geographical regions. The prevalence of chronic respiratory disease is 13.2% in the Americas, 12.4% in Europe, 13.5% in Asia, 13.9% in Africa and 11.6% in Oceania.^16-18^ The prevalence rate of COPD at the global level is estimated to be 12.6 percent.^19^ Surveys conducted in Iranian provinces have revealed a total COPD prevalence rate of 5.57 percent.^20^

Diabetes is the other chronic that has become a growing epidemic in the recent century.^21^ The recent estimates show that the global prevalence of diabetes in 2019 has reached to 9.3 percent (463 million persons) that will increase to 10.2 percent (578 million persons) in 2030, and 10.9 percent (700 million persons) in 2045.^22^ The prevalence of diabetes in Iran is also estimated to be 7.4-10.2 percent, and it is anticipated that 9.2 million Iranians will distress diabetes by 2030.^23,24^ The National Document for Prevention and Control of NCDs in Iran has also noted the four NCDs highlighted by WHO.^25^

In a source review, it was revealed that at the end of the twenty first century, the rate of disasters has increased globally 6 times that in 50 years ago.^26^ Therefore, human populations especially NCD patients are considered as vulnerable population in disasters.^27-31^ Iran is also among the 10 countries in the world due to its vulnerability to accidents and disasters, it has also been witnessing a rise in the prevalence and exacerbation of NCDs including diabetes and chronic respiratory diseases compared to other lower to middle income countries.^32^ Notwithstanding, COPD has not yet been integrated into the Package of Essential Non-communicable Disease Interventions (IraPEN) in Iran’s primary healthcare system. IraPEN plays an important role in directing, strengthening and improving the accountability of health care in non-communicable diseases in Iran.

Since the healthcare system is a multifactor system that is sensitive to cultural, economic, and sociopolitical conditions of a country, qualitative studies play an important role in the deep perception and understanding of people’s experiences on a certain phenomenon, and discovering the truth as it is experienced in research models.^37^ Therefore, the purpose of this study is to identify the problems of patients with NCDs in accidents and disasters from the perspective of People with chronic diseases, their families and specialists.

 Although different studies with qualitative and quantitative methods are conducted on the impacts of disasters on health needs of People with chronic diseases, the early warning system, and the preparedness of this vulnerable groups, a comprehensive qualitative research with a focus on diabetic and chronic respiratory disease patients has not yet been performed to identify challenges in providing healthcare services during disasters.^13,33-36^ Since the healthcare system is a multifactor system that is sensitive to cultural, economic, and sociopolitical conditions of a country, qualitative studies play an important role in the deep perception and understanding of people’s experiences on a certain phenomenon, and discovering the truth as it is experienced in research models.^37^ Therefore, the purpose of this study is to identify the problems of patients with NCDs in accidents and disasters from the perspective of People with chronic diseases, their families and specialists.

## Methods 

This study is a conventional content analysis (CCA) based on experts’ and People with chronic diseases’ experiences. Content analysis is a widely used qualitative research technique. In conventional content analysis, coding categories are derived directly from the text data. This approach is helpful when investigators aim to view participants’ experiences.^38^ In applying CCA, data is collected directly and without presumptions. Then codes, subcategories and categories are extracted via an inductive process.

**Participants**

In this study, purposeful sampling was applied to select participants. Due to the nature of NCDs and natural disasters, experienced and informed people in responsible organizations were investigated. Participants were selected in two expert levels including policymakers, executive managers, and university teachers experienced in NCD management in disasters; and People with chronic diseases include diabetes and chronic respiratory disease that were affected by natural disasters. This study was conducted in regions of Iran affected by earthquakes including Ahar, Varzaghan, Haris, and Sare-pol Zahab. Specifications of participants were shown in Table 1. 

**Table 1:** Specifications of patients and experts participated in the study of exploring challenges in providing care to patients with chronic diseases during disasters focusing on diabetic and chronic respiratory disease patients.

	Variable	Description	Number(Person)Relative Frequency (%)
	**Sex**	Male	11 (24%)
		Female	35 (76%)
		30-40	2 (4%)
	**Age**	41-50	5 (11%)
		51-60	14 (30%)
		61 and Up	25 (55%)
		Diabetes	31 (67%)
**Patients’**	**Type of Disease**	COPD	10 (22%)
		Diabetes and COPD	5 (11%)
	**History of Disease**	Less than 5 Years	11 (24%)
		5-10 Years	25 (54%)
		Over 11 Years	10 (22%)
		Illiterate	25 (54%)
	**Educational Level**	Junior High School Diploma	18 (39%)
		Senior High School Diploma	3 (7%)
	**Interview Method**	Face-to-Face	46 (100%)
		Phone Call	0
		**National and Provincial Policymakers**	18 (50%)
		NCD Specialists (Heads of centers for non-communicable diseases of the Ministry of Health and Medical Education), university teachers, EMS managers, MOHME managers, Red Crescent managers, disaster risk reduction and crisis managers (disaster managers of the province), university healthcare deputy, university health deputies	
	**Experience Level**	**Key Executive Authorities**	18 (50%)
		hospital healthcare deputy, pulmonologists and endocrinologists, hospital presidents, nursing personnel, health workers, nutrition experts, doctors and specialists of health centers	
**Experts’ **	**Age**	30-40	11 (31%)
		41-50	17 (47%)
		51-60	8 (22%)
	**Sex**	Male	22 (61%)
		Female	14 (39%)
	**Interview Method**	Face-to-Face	34 (94%)
		Phone Call	2 (6%)
	**Work Experience**	5-10 Years	8 (22%)
		11-16 Years	8 (22%)
		17 Years and More	20 (56%)

**Data Collection**

To collect data, the semi-structured interview guide for qualitative interviews was used, and responses directed the procedure of subsequent interviews. Due to its flexibility, this type of interview is appropriate to deepen qualitative studies.^39^ Initial questions were formulated based on the study objectives and upon reviewing documents under supervision of experts. In the beginning of interviews, research objectives, the method of interview, the interviewees’ right to participate or not participate in the study are explained by the investigator to participants. Interviews began with a general question and then entered into more detailed ones. Questions for the two groups of experts and People with chronic diseases were different and were developed based on challenges in providing care to diabetics and patients with chronic respiratory diseases during natural disasters as well as major challenges faced by these patients during earthquakes. For instance, experts were questioned of how they had managed diabetics and COPD patients during disasters, and what challenges they had faced. Or patients were questioned of their problems and their needs during disasters. After the first interview and having identified primary codes, other participants were selected based on newly extracted concepts. To select participants, purposeful sampling was applied, and to ensure that the number of participants was sufficient, the data saturation criterion was used.^40^ In a word, participants were selected from individuals with a good deal of information on the core question and the study objective. As a result, every identified concept was discussed in interviews unless saturated concepts and new information were produced. When the research reached a point, where no new aspects and characteristics were added to categories, and no data saturation and new link between categories revealed based on requirements, the sampling continued.^40^ Therefore, every identified concept was discussed in interviews until no new data was produced. 

All participants cooperated well in interviews by the investigator, and there were finally 36 participants from experts and 46 from diabetes and COPD patients eligible to enter the research. All subject People with chronic diseases had been citizens of the subject research environment for years and were living there; and for the purpose of an integrated management and prior coordination, the interviews were conducted at village health care centers; and for some elderly disabled patients interviews were conducted at their residence upon their permission. Regarding the subject experts, some were executive managers and university teachers who were citizens of the subject research environment, and some were policymakers, who resided in Tehran, capital city of Iran, and interviews were conducted in person at the workplace of each expert. 

EGh attended the field twenty-one times. Initially an interview was administered after the East Azerbaijan earthquake in August 11, 2012 in Ahar, Varzaghan, and Haris. Based on data needs, a subsequent coordinated interview was required to be administered for new experiences after the Kermanshah earthquake in November 12, 2017. Next, for the purpose of data completion, an interview was also administered following the earthquake in Sarre-pol Zahab, Kermanshah in November 25, 2018. Upon many attendances in all subject regions, totally 46 diabetic and COPD patients, and 36 experts were interviewed. 

27 interviews with patients were performed in Persian, and 19 interviews were performed in Kurdish native language. All interviews administered in Kurdish were translated by a translator fluent in both Persian and Kurdish, and familiar with basics of crisis management and first aid. The principal investigator (PI) was specialized and familiar with diabetes and chronic respiratory diseases, and with health in emergencies and disasters as well. Face to face interviews took 10 to 30 minutes with People with chronic diseases, while they took 15 to 90 minutes with experts. Interviews were performed face to face with one interviewee at a time. Only two of interviews with experts were administered by telephone, since one interviewee was on maternity leave and the other was on out-of-province business trip. Data collection was based on interviews and observations. 

**Data Analysis**

Data collection and analysis were carried out simultaneously. Upon the collaboration of the research team on the manner of selecting participants, all interviews were recorded digitally and in full, and they were transcribed word by word by Elham Ghazanchaei (EGh) as the PI. Before coding, the very interviews were read and listened several times, and the overall perception of the investigator was transcribed in about one page after each interview. To analyze data, Graneheim and Lundman method was applied. ^41^ Line by line interview continued for the identification of the condensed meaning units. Ultimately, the process of analyzing ended up by condensing meaning units to codes, primary subcategories, and main categories. According to the Principle of Similarity, similar codes were clustered together, and then similar subcategories were placed under certain categories. The data gathering and analysis process, and interviews were administered simultaneously. Data analysis was performed under close supervision of Davoud Khorasani Zavareh (DKZ) and Kiyoumars Allahbakhshi (KA). Encoding and categorizing the concepts were performed manually using paper and pencil. The research team aimed to acquire a deep perception of concepts, and avoid superficial coding. To ensure reliability, the four strategies of Lincoln and Guba were adopted.^42^

## Results

Specifications of the participants in the study are shown in Table 1. 

In this study, the mean age of subject diabetic and COPD patients was 50.71 years old. The standard deviation of the age of patients was ±10.72. The average history of disease in participants was 8.93 years with the standard deviation of ±5.41. On the other hand, the mean age of experts participating in this research was 44 years with the standard deviation of ±6.97, where the youngest age of the experts participating in the study was 30 years and the oldest age was 54. The average work experience of subject experts was reported to be 17.77 years with the standard deviation of ±7.62. 

2642 codes from interviews with patients with diabetes and chronic respiratory diseases, and 3171 codes from interviews with experts were obtained, and totally (People with chronic diseases and experts) 5813 codes were extracted. Having removed redundant cases and integrated similar ones, 159 patient codes and 306 expert codes and totally 465 (patient and expert) unique codes were obtained. In the next step, 46 main subcategories and 12 categories were developed. In the final step after the integration, challenges faced by COPD and diabetic patients were categorized based on participants’ (patient and expert) opinions in 4 categories and 12 subcategories: integrated management (with three subcategories of control and supervision, patient data management, and volunteer management), physical psychosocial health (PPSH) (with three subcategories of psychological impacts, exacerbation of signs and symptoms, and special patient characteristics), health literacy and the behavior (with three subcategories of risk perception, values and beliefs, and education and awareness) and barriers to healthcare delivery (with three subcategories of facilities and human resources, financial and living problems and insurances, accessibilities and geographic access). Categories, subcategories and codes are presented in Table 2 . Comments by experts are indicated within parentheses with letter E denoting Expert and comments by patients are indicated within parentheses with letter S denoting Sick. Strategies to acquire trustworthiness in this qualitative study by applying the Four-Dimension Lincoln and Guba’s Evaluative Criteria are presented in Table 3. 

**Table 2:** The content extracted from the qualitative research on challenges in providing care to diabetic and COPD patients during disasters.

Categories	Subcategories	Codes
		1) Lack of a systematic patient care pattern
	Control and Supervision	2) inadequate follow-up of patient treatment process
		3) Inter-organizational
		1) Lack of intelligent systems for identification of patients
Integrated Management	Patient Data Management	2) Loss of patient medical records
		3) Lack of private sector patient data
		1) Weak healthcare volunteer management
	Volunteer Management	2) Lack of job description for volunteers in disasters
		3) doubled work load of indigenous workers living in the region
		1) Patients’ grief and mourning
	Psychological Impacts	2) Patients’ stress and PTSD
		3) Patients’ fear and panic
		1) Blood sugar fluctuations
Physical Psychosocial Health (PPSH)	Exacerbation of Signs and Symptoms	2) Exacerbation of respiratory symptoms
		3) Neglecting late complications
		1) Being a single parent
	Special Patient Characteristics	2) Senility and disability of the patient in self-care
		3) Distressing underlying diseases
		1) Lack of early intelligent warning system for patients with chronic diseases
	Risk Perception	2) Lack of determining safe places in rural areas
		3) Neglecting the screening of patients with chronic medical disorders
		1) Fatalism
Health Literacy and the Behavior	Values and Beliefs	2) disrespect patients with chronic diseases
		3) Opportunism and business-mindedness of authorities in crises
		1) Low knowledge of service providers on helping patients with chronic diseases
	Education and Awareness	2) Neglecting scholars in crises by authorities
		3) Lack of personal preparedness in emergency evacuation
		1) Medicine shortage
	Facilities and Human Resources	2) Inadequate accommodation
		3) Lack of specialized health centers for patients with chronic diseases in disasters
		4) Lack of specialized medical staff
		1) Financial inability of patients in paying treatment costs
Barriers to Healthcare Delivery	Financial and Living Problems and Insurances	2) Increased out-of-pocket treatment payments
		3) Disability and unemployment
		4) Inadequate coverage of insurance organizations
		1) Overpopulation
	Accessibilities and Geographic Access	2) Border Inhabitation Issues
		3) Impassability of rural access roads

**Table 3:** Strategies to acquire reliability in the study to explore challenges in providing care to patients with chronic diseases during disasters focusing on diabetics and chronic respiratory disease patients in Iran.

Reliability Criterion	Description
**Credibility **	-The PI being immersed in data as well as findings of observations.
	-Allocating an adequate time of over 3 years for the gathering and interpretation of research data.
	-Attending consecutively the research environment in earthquake zones of Ahar, Haris, Varzaghan in East Azerbaijan province and in earthquake rural zones of Sare-pol Zahab in Kermanshah province. Over 10 village and healthcare centers.
	-Use of peer check technique by other research team members through the interactions of colleagues, questioning and answering and views.
	-Use of member check by participants in the study through re-interviewing 3 participants of experts group and 4 participants of patients group to support research findings.
	-Use of 2 experts out of the research team to examine questions and validate them (Expert Check).
	-Asking participants of any possible questions, ambiguities and misunderstandings during the interviews by EGh.
	-Gather data from different individuals involved, including diabetics and chronic respiratory disease patients involved in the crisis, experts and policymakers.
	-Use of numerous techniques to collect data besides interview, producing data through observation and search for contradictory evidences.
**Confirmability **	-Avoiding biases and remaining impartial in different subjects throughout the research process, writing memos and reports with verbatim quotations in the study.
	-Availability of all research documents including manuscripts.
**Transferability**	
	-Presenting exact details of participants in terms of their age, sex, education level, occupation, type of disease.
	-Presenting adequate information of the investigator, literature review, study process, type of participants, subject area.
**Dependability**	-Providing other researches with accurate information of the research process.
	-Use of accurate quotations of participants.
	-Audio recording, taking notes and recording other entries.

**Integrated Management**

**Control and Supervision**

Participants stated that manager’s first priority in crises was focused on communicable diseases, and patients with NCDs including diabetics and chronic respiratory disease patients were ignored in disasters; and that there was no systematic patient care pattern for such patients. Also, there was a weakness in the pursuit and the continuity of patient care after disasters, and in pre-incident planning. Moreover, there was no good estimation of proper distribution of resources and management of relief items, and there was a lack of coordination between organizations including Red Crescent, Municipality, Housing and Urban Development, Water and Wastewater, Ministry of Health, and Agricultural Jihad in patient management during disasters. Meanwhile, participants noted that no healthcare and treatment measures were taken for patients with chronic diseases in the first 2 weeks of the acute phase of a disaster, and this issue was not attended by authorities and directors of the country. They emphasized that there was no supervision by authorities over healthcare, treatment, and handling patients with NCDs in the country. 

*… The fact is that we have no formulated and systematic management system to intervene for non-communicable disease patients in the acute phase; and we have no plan either; and we do what the health system has already formulated as the patient care pattern (P26E)… I swear to God I managed my diabetes after the earthquake alone; and no patient care was provided by any authority or organization (P10S).*

**Patient Data Management**

According to participants, there was no accurate identification of diabetic and COPD patients prior to incidents, and patients did not introduce themselves to healthcare teams to be registered in a system titled as SIB System during the incident. It has been a few years since this system is developed in Iran wherein patient information is registered.^43^ They had also encountered different deficiencies including: a) unfamiliarity of users with the system, b) lack of access to the system by treatment deputies, and c) failure in system-related communication among university health and treatment deputies. Moreover, one challenge of the system was its failure to record information of patients with chronic diseases, and the lack of access to such information. Another challenge was the lack of information of patients, who were under care and treatment in private sectors. On the other hand, due to non-inclusion of chronic disease patients in the healthcare system of Iran, there was no accurate information of COPD patients.

*… They did not identify patients during the earthquake; and there was no monitoring of patients with health problems to be provided with healthcare (P6S)… Disasters had damaged infrastructures and communication systems; and patients’ medical records were damaged; and the lack of case-detection intelligent systems led to inadequate follow up and interruption in treatment procedures (P23E).*

**Volunteer Management**

From the viewpoint of some experts, a bad volunteer management in the crisis-zone was another existing challenge. After the Kermanshah earthquake, presence of many popular and executive forces at the place of the incident was not organized, and no job description was defined for them. The indetermination of non-local volunteers, and doubling the workload of indigenous volunteers were among other issues. On the other hand, non-local volunteers were not familiar with the region and failed to have a good verbal communication and conversation with patients. Excitement and sense-orientation in some popular volunteers were another challenges. Participants noted that a majority of volunteers had received no prior training. Among other existing challenges was the lack of monitoring volunteers’ performances to the extent that according to participants an inconsistency was evident in the distribution of volunteers in the region. 

*… Volunteers came from different cities; however, they were not organized at all, the hospital was crowded, and the presence of volunteers had caused interruptions in the work of medical personnel leading to disruptions (P29E)… Volunteer doctors were asking what to do. No clear job description was defined for volunteer aid workers during disasters (P26E).*

**Physical, Psychosocial Health**

**Psychological Impacts**

Based on participants’ experiences, one of the major challenges faced by patients after disasters was their psychological disorders. Some had experienced grief and mourning from the loss of their beloved ones and were in denial stage. Denying the truth was associated with the exacerbation of stress, anxiety and other incompatibility reactions. Participants acknowledged a tendency to smoke in patients with chronic respiratory diseases and diabetes. According to participants, service providers were as much affected psychologically as patients. On the other hand, failure to provide psychological counseling for diabetics and chronic respiratory disease patients was another challenge during crises. Weak psychological health policies for chronic disease patients during disasters and their low resilience were among major issues during the earthquake. Poor psychological support of managers and authorities were among other challenges noted by participants.

*… After the earthquake, I felt so bad. I always imagine the running out of the house, I can’t get to sleep at night or during the day. I have thought of suicide many times (P1S)… the earthquake fear and panic had made me mad, I was so much annoyed, living in tent was miserable, I lost many of my beloved ones (P16S).*

**Exacerbation of physical Signs and Symptoms of Disease**

From the viewpoint of participants, natural disasters had negative impact on clinical implications in diabetic and COPD patients. They acknowledged that the earthquake had caused the exacerbation of respiratory symptoms and shortness of breath in patients with chronic respiratory diseases; and that the blood sugar increased in patients with diabetes. Healthcare monitoring system outage during and after disasters, and disruptions in the continuity of health care were observed. Participants acknowledged that there was no monitoring over symptoms in patients with diabetes and COPD during and after the earthquake; and that no attention was paid to the oxygen level of patients with chronic respiratory diseases and their clinical conditions. According to participants, no patient test control was performed after disaster like what was done before the incident. The lung infections of chronic respiratory patients like pneumonia were among major challenges. 

*… After the earthquake, exposure to a huge amount of dust, caused my respiratory symptoms exacerbated; and I also experienced cardiac complications, and underwent angiography (P7S) … The stress caused impaired blood sugar in diabetic patients; and they sensed danger since their access to healthcare services was disrupted (P27E).*

**Special Patient Characteristics**

There was a general consensus among participants that patients with chronic diseases like diabetes and chronic respiratory diseases are among vulnerable groups in every society during disasters; and it is crucial to serve these people in the face of disasters since these diseases are known as silent killers. It is very important to identify and evaluate special characteristics of these patients and the health needs of these vulnerable populations during and after disasters. Senility and single parenting are examples of special characteristics of these patients that threaten their lives. Participants stated that another challenge at the time of earthquake is pregnant women with diabetes. Illiteracy of patients and their poor self-care were other major problems. According to participants, there was no attention paid to elderly patients with chronic diseases during disasters.

*… I’m distressing from COPD. Due to my unaffordability I could not buy aerosol sprays and oxygen cylinders. I have no caretaker and no salary, and I am not covered by any organization (P30S)… One challenge of management with respect to patients with diabetes and chronic respiratory diseases is that they are mainly elderly people not able to take care of themselves, and they have a systemic disease (P34E).*

**Health Literacy and the Behavior**

**Risk Perception**

Participants stated that the risk of the exacerbation of symptoms and impacts on the health status of patients with diabetes and chronic respiratory diseases was not well perceived. Moreover, authorities and managers did not place much emphasize on prevention strategies during and after natural disasters. They believed that in both Kermanshah and East Azerbaijan earthquakes, they were consistently witnessing a traditional approach and a lack of thinking of care for patients with chronic diseases. Lack of a safety management system for patients such as early warning systems was among challenges. Participants affirmed that authorities did not distinguish between the acute and normal state of patients with chronic diseases. They believed that a failure to include NCD patients in the National Disaster Preparedness and Response Plan was among major challenges. Failure to pre-determine safe zones for chronic disease patients during disasters was an important issue at the time of earthquakes.

*… With so many issues to be handled at the time of the earthquake, we as patients with chronic diseases were not a priority to authorities; no organization came to handle problems faced by patients. I had forgotten my illness myself, and nobody even came to me telling what to do (P28S)… The priority of authorities was to handle communicable- and traumatic diseases. Meanwhile, no place as muster point during disasters was designated for these patients (P22E).*

**Values and Beliefs**

Participants of this study acknowledged that during natural disasters like that of Kermanshah and Ahar earthquakes, where a vast area was affected, there were populations with different beliefs, cultures and views. On issue which was not attended by authorities in disasters like these earthquakes, were cultural-religious issues and dignity of people. According to participants, authorities’ negligence toward sociopolitical culture of these crisis-zones was among basic challenges. Some participants stated that they did not receive dispatched aids because they didn’t want to lose face, they felt shamed or because of they were single women. They noted that business-mindedness and opportunism of authorities were evident. On the other hand, participants stated that among challenges at the time of the earthquake was failure in acculturation of the importance of the place of EOC in relief organizations.

*… I have been distressing diabetes for years, and after the earthquake there was a rise in my blood sugar. I asked nobody for help. I am 70 years old, and I have never had ill-gotten and unlawful gains in my life, and have never asked anyone for food and medicine, and have never tried to catch trucks with relief goods to receive aids (P10S)… One of our leading weaknesses in the crisis was the opportunism and profit-mindedness of some authorities. They gave one snack bar to an earthquake victim and publicized it on the internet with 200 photos to showoff (P26E).*

**Education and Awareness**

According to participants, chronic disease management in the country is not well-prioritized during disasters. Patients and health care systems have no educational plans and no prior preparations to tackle and manage the crisis risk. Participants stated that the issue of patients with chronic diseases in disasters is only well-written down on paper, yet at the time of the incident, there is no preparation and coordination whatsoever while it is required for both service providers and patients. Participants claimed that crisis experiences and lessons are not used properly in the national guidelines for health care practice. Meanwhile, participants stated that they had received no training on self-care during disasters, and had no prior preparation. They stated that training to service providers had shown little effectiveness; and that there was no control over its effectiveness.

*… More important than the education is the use of experiences and lessons by those who had faced crises. It is important to train the service providers, doctors and patients. Trainings have not been perpetual and uninterrupted, and so has little effect on individual’s attitude (P18E)… When Ahar and Haris earthquakes of East Azerbaijan occurred, patients had not received any prior training… A patient who is illiterate does not know the name of the drug, does not know his/her own blood glucose level, and has not received any prior training, and misleads the physician’s diagnosis (P9E).*

**Barriers to Healthcare Delivery**

**Facilities and Human Resources**

Another issue that participants were concerned about was the supply of facilities, equipment, and human resources. They acknowledged that one of the most important challenges during Kermanshah, Varzaghan, Ahar, and Haris earthquakes was the scarcity of medications particularly diabetes medications including insulin and chronic respiratory disease medications including the spray. They were also faced with the shortage of oxygen cylinders for patients with respiratory diseases. The shortage of glucometers for measuring the blood glucose of diabetic patients was another problem. Also, other challenge existed during earthquakes was the failure in providing healthy diet for diabetic patients. Lack of health facilities and specialized medical centers for patients with chronic diseases was another challenge at the time of the earthquake. Moreover, at the time of Kermanshah, Varzaghan, Ahar, and Haris earthquakes, there was a lack of specialized workforce including social workers, pulmonologists, endocrinologists, physiotherapists. The number of advanced centers for patients with chronic respiratory diseases was small and hospitals were encountered with the lack of hospital beds for patients with chronic diseases, lack of ventilators, lack of laboratory and imaging services. 

*… When earthquake occurred, my house was destroyed, and dust annoyed me. I needed medications. I had no drugs for one week. I got lung infection but there was no pulmonologist available. There was no heating system. We made fire, and the smoke worsened my lung condition (P45S)… Among other challenges was the lack of a quite environment for the provision of care to patients with chronic diseases during disasters where they can be spiritually and physically supported (P29E).*

**Financial and Living Problems and Insurances**

One of the most fundamental challenges stated by participants was the natural disaster damage and the loss of patients’ assets, house demolition, loss of livestock and people’s livelihood. Many of patients lost their jobs after the earthquake. The earthquake caused the economic and living condition of the people of that region deteriorating; and they could not afford to pay their medical costs. It requires abundant financial resources to implement the post-disaster recovery plan for vulnerable groups like patients with chronic respiratory diseases and diabetes. Among other issues mentioned by participants were the inadequate coverage of insurance organizations, and the lack of a special guideline by insurance organizations for crises. Moreover, out-of-pocket payments of patients had increased. 

*… In crises and natural disasters, bodies such as supporting organizations like basic insurances do not modify their emergency protocols and procedure very much. Other supporting Organizations such as the Relief Committee and the Welfare Organization claimed to be out of budget meaning that their situation during emergencies would be the same as that in regular working conditions; and there would be no extra budget allocation for emergencies (P25E).*

**Accessibilities and Geographic Access**

Participants mentioned their limited access to medical centers stating that impassable access roads of their villages to medical centers were an important challenge at the time of earthquake. Moreover, insufficient attention of authorities to cross-border regions and marginalized urban areas were among major challenges faced by patients. On the other hand, roads were damaged by the earthquake which made the access to medical centers and laboratories difficult. Lack of specialized medical centers for patients with chronic diseases, and lack of rural health centers, had caused difficulties with the increased burden of patient visits to hospitals. The congestion and the problem of transporting patients with chronic diseases during crises were among other challenges after Kermanshah and East Azerbaijan earthquakes. Another issue discussed was the negligence by the municipality regarding the crowd management in the affected area. 

*… Since our village was distant, no doctor came to visit me. And I didn’t care anymore to go anywhere to see a doctor (P7S)… Due to damages following the earthquake, roads were impassable. Patients with chronic respiratory diseases were in need of chest x-ray, but since we had only one hospital which was destroyed, it was not possible to get the chest x-ray (P30E)*

## Discussion

The leading challenges in providing care to diabetic and COPD patients during disasters as discussed here include: the insufficient control and supervision, patient data management, volunteer management, values and beliefs, education and awareness, and shortage of facilities and human resources. 

Findings, based on experiences of participants, revealed that the inadequate control and supervision is one of the most important effective causes, and basic challenges in the integrated management of patients with diabetes and chronic respiratory diseases during disasters. Based on experiences of participants, one of the main causes is the central focus of responsible bodies on the management of communicable diseases in disasters, while chronic diseases including diabetes and COPD are often ignored in disasters. According to participants, among other causes of defective control and supervision, are the lack of an inter-organizational inconsistency during disasters, and the sense of superiority of one organization over others, which leads to an imperfect team working during disasters. Such inconsistencies among organizations in crises cause disruptions in the emergency response to diabetic and COPD patients; and it is among major reasons for the abandonment of patients with chronic diseased at the time of disasters. Another cause of the defective control and supervision, as pointed out by participants, is the negligence of authorities and policymakers concerning the deprived villages and cross-borders.

The lack of a systematic patient care pattern and deficiency in following-up their treatment process during and after disasters are among the major causes of inadequate control and supervision of diabetic and COPD patients in disasters, according to participants. Previous studies also revealed that natural disasters impact vulnerable populations such as patients with diabetes and COPD; and they considered the failure in timely provision of special care to patients, which required a control and supervision over policymakers and healthcare providers to improve their conditions in disasters, as the major cause of inadequate control and supervision over patients during disasters. Moreover, the existence of disease monitoring systems and training programs and development of resilient health systems are effective in reducing the challenge.^44-46^ According to findings of other studies, factors affecting the control and supervision of patient management during disasters include: failure to include NCDs in the health system policies, lack of guidelines on NCD clinical management in emergencies, and inconsistency among organizations in providing healthcare services, which has led to disruptions in systemic therapy of patients.^27,47^ Hence, according to participants, the coordination among sectors and promoting teamwork of organizations can play an effective role in the control and supervision of patients with chronic diseases during disasters. Moreover, consistent provision of healthcare services to patients after disasters and not forgetting them in crises are among operational suggestions. Participants acknowledged that development of job descriptions for every single relief organization for the purpose of managing chronic disease patients in disasters, and providing special care to such patients are important strategies. 

According to participants in this study, another basic challenge in providing care to patients with diabetes and COPD is the weak management of patient data during and after disasters. Based on their experiences, earthquakes caused the collapse of health homes and damaged patient medical records, and the lack of an intelligent system for patient identification was witnessed in the country. Another cause for the weak data management of diabetic and COPD patients in disasters, as noted by participants, was the lack of access to data and information of patients who had referred to private sectors seeking for healthcare services. Besides, failure in registering patients’ data in the national health care system was evident. Telecommunication service outage disrupted communications among organizations and communications with patients. Participants stated that factors including the outage of computer systems and the outage of Iran’s Integrated Health System entitled SIB were among other causes of disruption in patient data management during disasters. As a consequence, such disruption led to inaccurate estimation for the dispatch and distribution of human resources including doctors, nurses and other healthcare personnel to the affected area, and disruption in the supply of medication needs for chronic disease patients with diabetes and COPD as well. Other studies showed that the major causes of poor patient data management during disasters were the damaged medical records and damaged infrastructures following the incidents, which led to a failure in identifying patients, a disruption in their health care services, and an insufficient follow-up of their care process.^48-50^

Therefore, it is recommended to provide a safe system for patient identification. A patient data backup system during disasters will contribute to timely identification of patients.^35,51^ According to participants, generating patient electronic medical databases both in private sectors and in governmental units could be effective in identifying patients at the time of incidents and in timely provision of care to patients. 

Deficiencies in volunteer management during disasters are another basic challenge in providing care to patients with diabetes and COPD during disasters. Based on participants’ experiences, absence of job descriptions for volunteers, and the lack of trainings for volunteers before incidents were among the main causes of poor volunteer management.

Unfamiliarity of volunteers with the region, the culture and the language of patients is another cause of deficiencies in their management. Based on participants’ experiences, the absence of volunteer management caused deficiencies in their training, and the lack of training for volunteers had led to their emotion-oriented and sense-oriented behaviors during crises. On the other hand, the unfamiliarity of volunteers with patients of the region, their culture and colloquial language was another challenge argued. Participants stated that failure in volunteer management had led to improper distribution of work and doubled workload of indigenous workers, and even the conflict between indigenous and non-local volunteer forces. Other studies showed that poor volunteer management in the incident command system, is often caused by the absence of local emergency planning, and the lack of pre-training, since training required for volunteers before crises, and development of relations between volunteer organizations and universities can be effective.^52,53^

Another important finding of this study regarding challenges in providing care to patients during disasters is the inadequate attention paid to the issue of values and beliefs of patients in disasters. Based on participants’ experiences, authorities do not value chronic disease patients in disasters and their negligence to prioritizing patients is a major cause of neglecting the cultural issues as well as the dignity of patients. As part of the Sendai Framework for Disaster Risk Reduction 2015-2030, the first priority is that disaster risk management policies must be focused on a comprehensive perception of disaster risk.^54^

Diabetic and COPD patients participating in this study did not ask for aids from relief forces due to their religious and cultural beliefs. Among their reasons, they pointed to their fear of losing face, their feel of shame asking for help, and from a religious perspective they viewed asking for help as an unlawful act. Among other causes were the inadequate accountability of some personnel and the opportunism of authorities. Many of patients participating in this study deemed crisis as a type of fatalism and refused to receive help. Other studies showed that ignoring cultural issues lead to disruption in the reduction of disaster risk. Studies revealed that inadequate attention to local culture, and failure in teaching the regional native culture in providing care during disasters end up in cultural challenges.^55,56^

Meanwhile, in a study by Ardalan et al. which was conducted in Iran, it was revealed that chronic disease patients need attentions to their self-esteem, and necessity of developing cultural programs, and respect of their rights in disasters. Therefore, in consideration of different cultural and ethnic patterns in various countries on the one hand, and the impact of cultures on disaster risk perception and acceptance on the other, policymakers and planners are required to upgrade health plans and chronic disease patient management in disasters with regard to cultural norms, local history and regional customs.^55-57^

Deficiencies in education, awareness and preparedness of patients and service providers are among other major challenges identified in this study. According to participants’ views, one major cause of weakness in education and awareness of patients was the failure to present adequate educational materials to patients with diabetes and COPD, and their families when teaching them self-care during and after natural disasters. 

Moreover, according to the participants’ experiences, one cause of inadequate education and awareness of service providers was the lack of knowledge on managing these patients in disasters, and the unfamiliarity of healthcare providers with their own job description at the time of disasters. Other causes included the lack of preparedness in medical centers for providing care to patients with diabetes and COPD, and the lack of preparedness in relief organizations. Weak practice of pre-incident maneuvers and negligence of authorities in prevention and preparation issues were other causes argued by participants. In previous studies it was declared that the insufficient awareness of patients of the evacuation routs, and lack of an emergency plan to direct decision-making at the time of evacuation in crisis, are causes of inadequate education, awareness and preparedness.^58-60^

Defects in planning for preparation for healthcare provision was another cause as discussed in studies.^45^

The study conducted by Satoh et al. in Japan revealed that the promotion of preparedness in diabetic and COPD patients through training patients and health care providers causes the quick response to patients, and prevents their lives from being threatened.^61^ Moreover, it recommended emphasizing on medical curricula on disaster management, and the use of educational programs in the management of pulmonary and diabetic diseases.^62,63^ Previous studies recommended that community prepara-tion and response immediately after disasters, and elim-ination of inefficient strategies in the management of chronic disease patients can play an effective role in the support of this vulnerable group during disasters.^33,64^

One of the main priorities in Hyogo Framework for Action is the attention of states in using knowledge, innovation and education to build a culture of safety and resilience at all levels.^65^ Blaschke et al. noted that educating and managing high risk groups such as children, the elderly and chronic disease patients including patients with chronic respiratory diseases should be the first priority before disasters.^66^ Hence, measures like general and specialized education, practicing maneuvers, holding practical workshops, inclusion courses of chronic disease patient management in disasters in students’ curricula, practicing maneuver, and generating personal preparedness such as the emergency evacuation during disasters for patients with chronic diseases are recommended. Another finding of the study is the challenge of the lack of facilities and human resources from participants’ point of view. Participants stressed that the absence of specialized medical centers for chronic disease patients in disasters caused disruptions in the consistency of health care and treatment of patients. Many of these patients were unable to self-care due to their senility. Because of emergency conditions and outage of power, lack of medical refrigerator, and scarcity of oxygen, these patients were facing difficulties. The oxygen-dependent COPD patients distressed from the exacerbation of respiratory distress signs due to impaired access to oxygen and respirator masks during disasters. Barriers to provide care to patients threatened the life of chronic disease patients in the research subject crises. Moreover, the lack of human force including doctors, nurses, physiotherapists were among other causes of resource scarcity. According to previous studies, patients with diabetes and COPD faced difficulties of supplying medicine including insulin, scarcity of respirator masks, uncertainty of their ability to take medication and non-adherence to diet.^47,67,68^

Based on participants’ experiences, financial inability to pay medical costs, and the inadequate coverage of insurance organizations during disasters led to non-referral of patients to medical centers, and adhere to self-care behavior, which was life threatening in disasters. Lack of accurate patient identification and screening in crises, and scarcity of health care facilities including laboratories and imaging services caused disruptions in the continuity and follow-up with patient care. The food insecurity threat, shortage of good heating systems, scarcity of respiratory protective equipment for diabetic and COPD patients were among other challenges argued by participants. Previous research also found that the lack of facilities including the issue of medication supply and insufficient attention to proper diet for chronic disease patients during and after disasters are critical challenges that lead to a delay in the treatment of patients.^46,67,68,69^

Therefore, participants strictly recommended pharmaceutical and healthcare service access, and door-to-door service monitoring even in impassable affected areas. They stated that the access to safe drinking water and food is one effective solution. Participants suggested the development of a telemedicine system, and providing timely access to healthcare services in a place allocated to this patient.

**Strengths and Weaknesses of the Study**

This study is conducted as a response to public health concern regarding the health outcomes in patients with chronic diseases including diabetes and COPD during disasters, and the challenges they face in natural disasters. With the information obtained from participants, we were able to identify challenges, which can improve and strengthen preparedness for disasters, and the disease management for diabetic and COPD patients; and consequently promote the outcomes of healthcare provided to these patients. This study is among few studies (perhaps the second or third study) that have employed qualitative method to obtain experiences of both diabetic and COPD patients, and main experts regarding the challenges in providing care to patients during disasters. Participants in this study were patients who have been for years distressing from diabetes and COPD; and have experienced disasters and experts, who have enough experience and knowledge, and have been present in all provinces of the country, and are experienced. Strength of this study is data collection through several cases of incidents in different provinces around the country. In this research, some authorities did not participate because they were either too busy or were not available. Notwithstanding, the research team tried to saturate concepts by other experienced participants. In future studies, authors can investigate patients with other chronic diseases in similar countries, and then compare the results with this study and evaluate them.

## Conclusion

This study is among few studies that have employed qualitative method to extract experiences of both patients and experts regarding the challenges in providing care to chronic disease patients during disasters. Findings of this research can facilitate to direct and support the planning, and the provision of quality, safe, efficient and effective healthcare services to diabetic and COPD patients during disasters. In consideration of the global increasing prevalence of chronic diseases on the one hand, and the growing global incidents and disasters, and the vulnerability of chronic disease patients in disasters, findings of this study may be helpful and applicable in other communities. The increased disasters and the chronic disease epidemic particularly that of diabetes and chronic respiratory diseases may be controlled and prevented by identifying challenges, and applying suggested solutions in this study. We can conclude by findings of this study that the needs assessment at the time of natural disasters requires a comprehensive approach to protect people with diabetes and COPD, who are facing numerous health care issues.

The principal addressees of this study are patients with diabetes and chronic respiratory diseases who may be engaged every moment in a natural disaster like flood, earthquake, tsunami, etc. Moreover, other addresses are relief organizations including the Red Crescent, Ministry of Health and Medical Education, specialists and experts in various groups including doctors, nurses, assistant nurses, paramedics, and any planner and policymaker in the health care system. Lack of control and supervision by responsible bodies and their central focus on communicable disease management has caused disruptions in the integrated management of chronic disease patients during and after disasters. Hence, paying attention to the management and training issues of patients and service providers, and paying attention to patients’ value and belief patterns in disasters will help resolving challenges. Findings of this study revealed some important and integrated information regarding the challenges in providing care to patients with diabetes and chronic respiratory diseases in disasters; and efforts can be made through strong countermeasures against health and monitoring system outage to identify needs and health problems of this group of patients, and prepare them for the future disasters. 

**Acknowledgement**

This study is part of a doctoral dissertation carried out by the Social Determinants of Health Research Center of Urmia University of Medical Sciences in Iran with Ethical Code IR.UMSU.REC.1398.228. Authors acknowledge and appreciate the participation of main experts in different provinces and patients from Kermanshah and East Azerbaijan who shared their experiences with investigators. 

## References

[1] Sleeman KE, de Brito M, Etkind S, Nkhoma K, Guo P, Higginson IJ (2019). The escalating global burden of serious health-related distressing: projections to 2060 by world regions, age groups, and health conditions. Lancet Glob Health.

[2] Organization WH. Global action plan on physical activity 2018-2030: more active people for a healthier world: World Health Organization; 2019.

[3] Idris IO, Oguntade AS, Mensah EA, Kitamura N (2020). Prevalence of non-communicable diseases and its risk factors among Ijegun-Isheri Osun residents in Lagos State, Nigeria: a community based cross-sectional study. BMC Public Health.

[4] Organization WH. Global tuberculosis report 2013: World Health Organization; 2013.

[5] Abegunde DO, Mathers CD, Adam T, Ortegon M, Strong K (2007). The burden and costs of chronic diseases in low-income and middle-income countries. Lancet.

[6] Mathers CD, Loncar D (2006). Projections of global mortality and burden of disease from 2002 to 2030. PLoS Med.

[7] Geneau R, Stuckler D, Stachenko S, McKee M, Ebrahim S, Basu S (2010). Raising the priority of preventing chronic diseases: a political process. Lancet.

[8] Organization WH. Global status report on noncommunicable diseases 2014: World Health Organization; 2014.

[9] Salam R (2016). Expanding the definition of noncommunicable disease. Journal of Social Health and Diabetes.

[10] Dye C. After 2015: infectious diseases in a new era of health and development. Philosophical Transactions of the Royal Society B: Biological Sciences. 2014;369(1645). 10.1098/rstb.2013.0426 PMC4024220 24821913

[11] WH O. Global action plan for the prevention and control of noncommunicable diseases 2013–2020. World Health Organization Geneva, Switzerland; 2013.

[12] Crettenden IF, McCarty MV, Fenech BJ, Heywood T, Taitz MC, Tudman S (2014). How evidence-based workforce planning in Australia is informing policy development in the retention and distribution of the health workforce. Hum Resour Health.

[13] Ryan B, Franklin RC, Burkle Jr FM, Aitken P, Smith E, Watt K, et al. Identifying and describing the impact of cyclone, storm and flood related disasters on treatment management, care and exacerbations of non-communicable diseases and the implications for public health. PLoS Curr. 2015 10.1371/currents.dis.62e9286d152de04799644dcca47d9288 PMC4593706 26468423

[14] Mannino DM, Buist AS (2007). Global burden of COPD: risk factors, prevalence, and future trends. Lancet.

[15] Organization WH. Chronic obstructive pulmonary disease (COPD). 2017, http://www who int/respiratory/copd/en. 2018, accessed 9 March 2021.

[16] Menezes AMB, Perez-Padilla R, Jardim JB, Muiño A, Lopez MV, Valdivia G (2005). Chronic obstructive pulmonary disease in five Latin American cities (the PLATINO study): a prevalence study. Lancet.

[17] Gupta D, Agarwal R, Aggarwal AN, Maturu V, Dhooria S, Prasad K (2013). Guidelines for diagnosis and management of chronic obstructive pulmonary disease: Joint ICS/NCCP (I) recommendations. Lung India.

[18] Blanco I, Diego I, Bueno P, Fernández E, Casas-Maldonado F, Esquinas C (2019). Geographic distribution of chronic obstructive pulmonary disease prevalence in Africa, Asia and Australasia. Int J Tuberc Lung Dis.

[19] Varmaghani M, Dehghani M, Heidari E, Sharifi F, Moghaddam SS, Farzadfar F (2019). Global prevalence of chronic obstructive pulmonary disease: systematic review and meta-analysis. East Mediterr Health J.

[20] Varmaghani M, Farzadfar F, Sharifi F, Rashidian A, Moin M, Moradi-Lakeh M (2016). Prevalence of asthma, COPD, and chronic bronchitis in Iran: a systematic review and meta-analysis. Iran J Allergy Asthma Immunol.

[21] Glovaci D, Fan W, Wong ND (2019). Epidemiology of diabetes mellitus and cardiovascular disease. Curr Cardiol Rep.

[22] Saeedi P, Petersohn I, Salpea P, Malanda B, Karuranga S, Unwin N (2019). Global and regional diabetes prevalence estimates for 2019 and projections for 2030 and 2045: Results from the International Diabetes Federation Diabetes Atlas. Diabetes Res Clin Pract.

[23] Wild S, Roglic G, Green A, Sicree R, King H (2004). Global prevalence of diabetes: estimates for the year 2000 and projections for 2030. Diabetes care.

[24] Esteghamati A, Larijani B, Aghajani MH, Ghaemi F, Kermanchi J, Shahrami A (2017). Diabetes in Iran: prospective analysis from first nationwide diabetes report of National Program for Prevention and Control of Diabetes (NPPCD-2016). Sci Rep.

[25] Peykari N, Hashemi H, Dinarvand R, Haji-Aghajani M, Malekzadeh R, Sadrolsadat A (2017). National action plan for non-communicable diseases prevention and control in Iran; a response to emerging epidemic. J Diabetes Metab Disord.

[26] Bronfman NC, Cisternas PC, Repetto PB, Castañeda JV (2019). Natural disaster preparedness in a multi-hazard environment: Characterizing the sociodemographic profile of those better (worse) prepared. PLoS One.

[27] Murakami A, Sasaki H, Pascapurnama DN, Egawa S (2018). Noncommunicable diseases after the Great East Japan Earthquake: systematic review, 2011–2016. Disaster Med Public Health Prep.

[28] Ochi S, Leppold C, Kato S (2020). Impacts of the 2011 Fukushima nuclear disaster on healthcare facilities: A systematic literature review. International Journal of Disaster Risk Reduction.

[29] Lami F, Jewad AW, Hassan A, Kadhim H, Alharis S (2019). Noncommunicable Disease Emergencies During Arbaeenia Mass Gathering at Public Hospitals in Karbala, Najaf, and Babel Governorates, Iraq, 2014: Cross-Sectional Study. JMIR Public Health Surveill.

[30] Ruby A, Knight A, Perel P, Blanchet K, Roberts B (2015). The effectiveness of interventions for non-communicable diseases in humanitarian crises: a systematic review. PLoS One.

[31] Akik C, Ghattas H, Mesmar S, Rabkin M, El-Sadr WM, Fouad FM (2019). Host country responses to non-communicable diseases amongst Syrian refugees: a review. Confl Health.

[32] Esteghamati A, Khalilzadeh O, Anvari M, Meysamie A, Abbasi M, Forouzanfar M (2009). The economic costs of diabetes: a population-based study in Tehran, Iran. Diabetologia.

[33] Gichomo G. Improving Disaster Preparedness and Planning for Chronic Disease Populations: Walden University ProQuest Dissertations Publishing; 2019.

[34] Slama S, Kim H-J, Roglic G, Boulle P, Hering H, Varghese C (2017). Care of non-communicable diseases in emergencies. Lancet.

[35] Aldrich N, Benson WF (2008). Disaster preparedness and the chronic disease nedds of vulnerable older adults. Prev Chronic Dis.

[36] Korteweg HA, van Bokhoven I, Yzermans C, Grievink L (2010). Rapid health and needs assessments after disasters: a systematic review. BMC Public Health.

[37] Corbin J, Strauss A. Basics of qualitative research: techniques and procedures for developing grounded theory, 2014.

[38] Elo S, Kyngäs H (2008). The qualitative content analysis process. J Adv Nurs.

[39] Speziale HS, Streubert HJ, Carpenter DR. Qualitative Research in Nursing Book; 2011.

[40] Glaser BG, Strauss AL. The discovery of grounded theory: Strategies for qualitative research; 2017.

[41] Graneheim UH, Lundman B (2004). Qualitative content analysis in nursing research: concepts, procedures and measures to achieve trustworthiness. Nurse education today. Nurse Educ Today.

[42] Schwandt TA, Lincoln YS, Guba EG (2007). Judging interpretations: But is it rigorous? Trustworthiness and authenticity in naturalistic evaluation. New directions for Evaluation.

[43] Jafari H, Ranjbar M, Amini-Rarani M, Hashemi FS, Bidoki SS (2020). Experiences and Views of Users about Delivering Services through the Integrated Health System: A qualitative study. The Journal of Tolooebehdasht.

[44] Hassan S, Nguyen M, Buchanan M, Grimshaw A, Adams OP, Hassell T (12). Management of Chronic Noncommunicable Diseases After Natural Disasters in The Caribbean: A Scoping Review: A scoping review of literature published between 1974 and 2020 examining the burden and management of chronic noncommunicable diseases after natural disasters in the Caribbean. Health Aff (Millwood). 2020.

[45] Allweiss P (2019). Diabetes and disasters: recent studies and resources for preparedness. Curr Diab Rep.

[46] Ryan B, Franklin R, Burkle F, Smith E, Aitken P, Leggat P (2019). Determining key influences on patient ability to successfully manage noncommunicable disease after natural disaster. Prehosp Disaster Med.

[47] Demaio A, Jamieson J, Horn R, de Courten M, Tellier S. Non-communicable diseases in emergencies: a call to action. PLoS currents. 2013 Sep 6;5. 10.1371/currents.dis.53e08b951d59ff913ab8b9bb51c4d0de PMC3775888 24056956

[48] Man RX-G, Lack DA, Wyatt CE, Murray V (2018). The effect of natural disasters on cancer care: a systematic review. Lancet Oncol.

[49] Gohardehi F, Seyedin H, Moslehi S (2020). Prevalence Rate of Diabetes and Hypertension in Disaster-Exposed Populations: A Systematic Review and Meta-Analysis. Ethiop J Health Sci.

[50] Ghazanchaei E, Khorasani-Zavareh D, Aghazadeh-Attari J, Mohebbi I (2022). Establishing the Status of Patients With Non-Communicable Diseases in Disaster: A Systematic Review. Disaster Med Public Health Prep.

[51] Pourhosseini SS, Ardalan A, Mehrolhassani MH (2015). Key aspects of providing healthcare services in disaster response stage. Iran J Public Health.

[52] Downey LH, Buys D, Fountain B, Ball T, Hilbun AH, Threadgill P. Assisting after Disaster: A Volunteer Management and Donations Management Training. Journal of Extension. 2018;56(3):n3.

[53] Kankanamge N, Yigitcanlar T, Goonetilleke A, Kamruzzaman M (2019). Can volunteer crowdsourcing reduce disaster risk? A systematic review of the literature. International Journal of Disaster Risk Reduction.

[54] Walch C (2015). Expertise and policy-making in disaster risk reduction. Nature Climate Change.

[55] Afrian R, Hariadi J, Akob B, Islami ZR, Local Culture Inventory for Disaster Mitigation Learning; 2020.

[56] Kulatunga U (2010). Impact of culture towards disaster risk reduction. International Journal of Strategic Property Management.

[57] Ardalan A, Mazaheri M, Naieni KH, Rezaie M, Teimoori F, Pourmalek F (2010). Older people's needs following major disasters: a qualitative study of Iranian elders' experiences of the Bam earthquake. Ageing and Society.

[58] Uscher-Pines L, Hausman AJ, Powell S, DeMara P, Heake G, Hagen MG (2009). Disaster preparedness of households with special needs in southeastern Pennsylvania. Am J Prev Med.

[59] Kang K (2014). Disaster preparedness among vulnerable older adults with chronic diseases: Results from a cross sectional study in Incheon, Korea. Nurs Health Sci.

[60] Owens JK, Martsolf DS (2014). Chronic Illness and Disasters: Development of a Theoretical Framework. Qualitative Report.

[61] Satoh J, Yokono K, Ando R, Asakura T, Hanzawa K, Ishigaki Y (2019). Diabetes care providers’ manual for disaster diabetes care. J Diabetes Investig.

[62] Koenig KL, Schultz CH, editors. Koenig and Schultz's disaster medicine: comprehensive principles and practices. Cambridge University Press; 2010.

[63] Robinson B, Alatas MF, Robertson A, Steer H (2011). Natural disasters and the lung. Respirology.

[64] Ghazanchaei E, Mohebbi I, Nouri F, Aghazadeh-Attari J, Khorasani-Zavareh D (2021). Non-communicable diseases in disasters: a protocol for a systematic review. J Inj Violence Res.

[65] Mirzaei S, Mohammadinia L (2019). Importance of Developing a Local Instrument of Disaster-resilient School: Letter to Editor. Journal of Community Health Research.

[66] Blashki G, Armstrong G, Berry HL, Weaver HJ, Hanna EG, Bi P (2011). Preparing health services for climate change in Australia. Asia Pac J Public Health.

[67] Mori K, Ugai K, Nonami Y, Kirimura T, Kondo C, Nakamura T (2007). Health needs of patients with chronic diseases who lived through the great Hanshin earthquake. Disaster Manag Response.

[68] Nakayama T, Tanaka S, Uematsu M, Kikuchi A, Hino-Fukuyo N, Morimoto T (2014). Effect of a blackout in pediatric patients with home medical devices during the 2011 eastern Japan earthquake. Brain Dev.

[69] Nguyen MN. Asthma-Related Health Outcomes in New Jersey After a Natural Disaster Event. Doctoral dissertation, Old Dominion University, 2019.

